# Bibliographical Notices

**Published:** 1848-11

**Authors:** 


### EDITORIAL DEPARTMENT.

### BIBLIOGRAPHICAL NOTICES.

*A Practical Treatise on the Causes, Symptoms, and Treatment, of Spermatorrhœa.* By M. Lallemand; formerly Professor of Clin. Surgery at the University of Montpelier, etc. Translated and edited by Henry J. McDougal, F. R. S., of England, etc. Philadelphia. Lea & Blanchard. 8vo. pp. 320: 1848.

If called upon to cite a book exhibiting, pre-eminently, the errors and evils of specialties, we know not that a better illustration could be found than is afforded by the above work. The author begins by submitting the details of five fatal cases, in each of which spermatorrhœa had either been ascertained, or suspected to have existed at a period more or less remote from the patient's death, and in which more or less disease of the genito-urinary apparatus was discovered at the autopsies. But in each case disease sof other organs existed sufficient to account for death, and the most prominent of the symptoms. That the latter affections originated consecutive to, and as consequences, of those appertaining to the genito-urinary apparatus, is a conclusion based on the merest hypothesis.

The same criticism will apply to a majority of the numerous cases which are contained in the work. The author assumes that spermatorrhœa, in whatever pathological connections it may obtain, is the essential primitive disease, as a matter of course, and, he appears sometimes to decide upon the presence of spermatorrhœa, inferentially, from the presence of phenomena which he has, in some cases, found associated with that symptom. The book, in short, shows that the author's mind is so imbued with the idea that the particular malady of which he treats is the *fons et origo* of a great proportion of the ills that "flesh is heir to," that it does not seem to occur to him to suspect that in some instances it may be merely a concurrent symptom, an epi- or anti-phenomenon, or an effect of the disorders which he attributes to it. In this he exemplifies, as already remarked, the dangers, and the evils of investigations limited, too exclusively, to special diseases.

The pathological effects of the disordered action, and of the voluntary abuse of the generative functions, constitute an important subject for medical researches. That it is a subject involving, in a certain sense, indelicate and disgusting details, does not make it less deserving attention in a scientific point of view, in as much as there is reason to believe that physical and moral evils of no small magnitude may be traced to this source, and might perhaps be averted, in some measure, were they better understood. The subject, in our opinion, has received too little consideration hitherto. Too little pains have been taken to ascertain facts; and there has been, not only too little attention to the acquisition of farther knowledge, but a degree of oversight and silence, as regards what is known, which is almost culpable. Our patients, and the public, have not derived that benefit from what information medicine already possesses on the subject, which they have a right to claim of the profession. Physicians have yielded too much to the repulsiveness of the subject. A portion, however, err in the opposite extreme. Some, like M. Lallemand, attach to this, as the source of physical and moral evils, by far too much importance. Not a little mischief sometimes, arises from this error, from its influence upon minds already affected with hypochrodriasis. Insanity, we believe to be frequently produced, not so much by the abuse, or pathological condition of the functions referred to, as by the melancholy conviction that irreparable injury has been inflicted upon the intellect and constitution, and the despondency and sense of degradation which grow out of this conviction. The harpies whose infamous business is to prey upon the credulity and fears of all who come within their grasp, have discovered this method of securing victims, and not a few instances have come within our knowledge in which sensitive persons have been tortured so long as their fears and credulity could be turned to profitable account.

But, returning from this digression to the work which it is our present business to notice, owing to the defects of which we have spoken, it possesses, pathologically, in our estimation, but little value, and is not calculated to shed much light on the morbid relations of the special disorder of which it treats. Therapeutically, it offers little that is new, except the surgical remedy which, we believe, originated with M. Lallemand, to wit, cauterization of the prostatic portion of the urethra. It is now several years since this measure was proposed by M. Lallemand, and it might reasonably be supposed that the collective experience of surgeons should, by this time, have determined its merits with some definiteness. So far as we know, the results of its application in the hands of others, have been, for the most part, negative. We are not surprised at this, for the measure has always appeared to us a mere novelty, which, like other novelties of the same sort, would be short lived. Whatever benefit may appear, in some instances, to be derived from it, may probably be explained by the moral and physical impression that such an operation is calculated to produce, irrespective of any appropriateness based upon rational, pathological grounds. We confess we have never made trial of it. Entertaining the views just expressed, we have preferred to await the results of the experience of others, before resorting to it in our own practice; and the consequence of this delay will probably be, that we shall, by and by, be hardly justified in resorting to it, should we happen to be disposed so to do.

In conclusion, we may remark, that we are unable to perceive that the work is calculated to do good except in so far as it may serve to direct the attention of the profession to an important and neglected subject, and, possibly, by its defects and errors, creating investigations which may lead to more useful results.

---

*New Treatise on Venereal Diseases.*—A volume has lately made its appearance in New York, entitled "*Nature and Treatment of Venereal Diseases,*" by an M. D., which for size and impudence will not soon be beaten. It has not yet appeared in our book-stores, and it probably intends to avoid the reviewer's notice, but it is circulated by a travelling agent whose business it is to impose upon such as do not recognize the cheat, and to receive, with well practised composure, the rebukes of those who understand the book and its author.

The work is a quarto of nearly 400 pages, and on excellent paper, illustrated with a large number of most beautifully executed, and finely colored drawings. But, to a very great extent, the plates are exact copies of a work published four or five years since in Paris, by M. Ricord, and entitled "Clinique Iconographique de l'Hopital des Veneriens," and the text is but a correct translation of his cases as recorded on the parallel pages of the work. These pictures and their corresponding descriptions being thus faithfully copied into this book of Dr———, without the slightest acknowledgement. The reader is therefore left to suppose that they are cases which have come under the care of Dr.———

Having ascertained thus much of the merits of the book, we were unwilling to examine farther, thinking that we should prefer the originals.

If we are not wrong in our divination, this Dr. was not long since a Dentist; and is now a licentiate from the county society of the city of New York, and is especially known in that city as the unblushing quack who publishes daily in the advertising sheets of certain city penny papers more wonderful cures and capital operations than are made by all the other doctors in the city of New York in a month. One need scarcely to have studied "Lichtenberg's Physiognomy of Cues," to have discovered the author's character, in the first pages of the book; and now that we have given the "cue," we trust none of our readers will be taken in. *Cave a signatis!*

F. H. H.

---

*The Lungs; their Uses, and the Prevention of their Diseases; with Practical Remarks on the Use of Remedies by Inhalation.* By James Stewart, M. D,; Fellow of the College of Physicians and Surgeons. Author of a practical treatise on the diseases of children, etc. New York. W H. Graham, Tribune Buildings. 1847: 18mo. pp. 139.

We cannot do better justice to this little book than by adopting the language of a friend whose opinion is worth much more than ours, and whose name (if we felt at liberty to give it in connection with a quotation from a private letter) would carry with it great weight. He says:—"I would call your attention to the merits of this little *brochure*, which, if I mistake not, you will find possessed of no common merit. It is really a philosophical and valuable little treatise, full of useful hints and suggestions, and worthy the particular attention of both laymen and doctors." We will only add, that if the reader of the book should not concur in the encomiums of our friend, he will have little to regret as respects loss of time or money, as its cost is a trifle, and it can be perused in an hour.

